# Molecular evolutionary dynamics of enterovirus A71, coxsackievirus A16 and coxsackievirus A6 causing hand, foot and mouth disease in Thailand, 2000–2022

**DOI:** 10.1038/s41598-023-44644-z

**Published:** 2023-10-13

**Authors:** Pirom Noisumdaeng, Pilaipan Puthavathana

**Affiliations:** 1 Faculty of Public Health, Thammasat University, Pathum Thani, 12120 Thailand; 2 Thammasat University Research Unit in Modern Microbiology and Public Health Genomics, Thammasat University, Pathum Thani, 12120 Thailand; 3 Faculty of Medical Technology, Mahidol University, Nakhon Pathom, 73170 Thailand

**Keywords:** Evolution, Genetics, Microbiology, Molecular biology, Diseases

## Abstract

Hand, foot and mouth disease (HFMD) is a public health threat worldwide, particularly in the Asia–Pacific region. Enterovirus A71 (EV-A71), coxsackievirus A16 (CVA16), and CVA6 are the major pathogens causing HFMD outbreaks in several countries, including Thailand. We retrieved 385 VP1 nucleotide sequences, comprising 228 EV-A71, 33 CVA16, and 124 CVA6, deposited in the databases between 2000 and 2022 for molecular evolutionary characterization using Bayesian phylogeny. All EV-A71 identified belonged to genotype B, subgenotypes B4, and B5, and to genotype C, subgenotypes C1, C2, C4a, C4b, and C5. The analyzes demonstrated these viruses’ co-circulation and subgenotypic changes throughout the past two decades. The CVA16 was grouped in genotype B1, predominantly subgenotype B1a, and the CVA6 was grouped in subgenotype D3, clades 1–4. The tMRCA of EV-A71 genotypes B and C, CVA16 B1, and CVA6 D3 dated 1993.79, 1982.62, 1995.86, and 2007.31, respectively, suggesting that the viruses were likely introduced and cryptically circulated in Thailand before the HFMD cases were recognized. We demonstrated these viruses’ fluctuation and cyclical pattern throughout the two decades of observation. This study provided insight into evolutionary dynamics concerning molecular epidemiology and supported the selection of current genotype-matched vaccines, vaccine development, and implementation.

## Introduction

Hand, foot and mouth disease (HFMD) poses a substantial public health threat worldwide, particularly in Asia and the Pacific region. The disease is common in infants and young children, but sometimes, it can occur in adolescents and adults^1^. The symptoms of HFMD in childhood include febrile illness with the papulovesicular rash on palms or soles, with or without ulcers in the oral cavity. Sometimes, the rash may appear on the buttocks, knees, and elbows, and it may be a maculopapular rash without vesicles. HFMD spreads from person to person by direct contact with respiratory secretions and fecal–oral transmissions.

HFMD is caused by multiple enterovirus A, of which enterovirus A71 (EV-A71) and coxsackievirus A16 (CVA16) have become the major causative agents for decades^2–6^. Some EV-A71-infected patients may present with cardiopulmonary and/or central nervous system (CNS) complications^7, 8^. Coxsackievirus A6 (CVA6) was isolated and characterized in the U.S. for the first time in 1949 without clinical information^9^. After Finland reported the outbreak in 2008, CVA6 became one of the major etiologic agents causing HFMD in several countries in Europe, North America, and Asia^10^.

HFMD was first noticed in Thailand in 2000, and it became a notifiable disease by the Ministry of Public Health (MOPH) in 2001^11, 12^. The first HFMD outbreak was reported in 2012 and attacked 45,464 patients^13^. The numbers of HFMD patients annually reported to the MOPH between 2000 and 2022 were sum-up in this study and made the cumulative numbers of over 720,842 cases with a total of 42 deaths (approximately 0.006% case fatality). Most of the reported cases (87.9–93.9%) are infants and children of age ≤ 5 years^11, 12^. The disease is highly prevalent in the rainy season, which lasts between May and October annually. Similar to what happened worldwide, EV-A71 and CVA16 have been the major causative agents of HFMD in Thailand during the last decades^4, 14, 15^. Complete genome analysis of the viruses circulated from 2006 to 2014 demonstrated that one out of 20 EV-A71 and all seven CVA16 analyzed were recombinant viruses^14^. CVA6 emerged and caused a large outbreak in Thailand in 2012^16^. After the outbreak subsided, CVA6 did not disappear but co-circulating along with EV-A71 and CVA16 up to the present^17, 18^.

EV-A71, CVA16, and CVA6 belong to the species *Enterovirus A* (EV-A), genus *Enterovirus*, in the family *Picornaviridae*. The viruses are small, non-enveloped with icosahedral symmetry, and contain a positive sense, single-stranded RNA genome of approximately 7.4 kb in length. The genome coding sequence was flanked with a 5′ untranslated region (5′UTR) linking to the virus-encoded peptide (VPg) and a poly-A tail at the 3′ untranslated region (3′UTR). The genome contains one open reading frame (ORF) with 3 regions: P1, P2, and P3. P1 encodes 4 structural proteins: VP1-4, P2 encodes 3 non-structural proteins: 2A-2C, and P3 encodes 4 non-structural proteins: 3A-3D^14, 19^. Based on the VP1 region, which is the most variable domain and major antigenic protein for inducing neutralizing antibodies, EV-A71 viruses are classified into eight genotypes (A–H)^2, 20–22^. Genotype A contains a single-member, BrCr prototype initially identified in a patient with encephalitis in California, USA, in 1969^23^. Genotypes B and C comprised multiple subgenotypes, B0–B5 and C1–C6^2, 15, 22^. Genotypes D and G were discovered in India from patients with acute flaccid paralysis, and genotypes E and F were discovered in Africa and Madagascar, respectively^20^. Genotype H was detected in sewage samples in Pakistan^21^. Similarly, CVA16 comprised four genotypes (A–D) based on VP1 or VP4 sequences^5, 6, 15, 24, 25^. Prototype G-10 is the only member of genotype A, while genotype B is further divided into subgenotypes B1 (B1a, B1b, and B1c), B2^5, 15^, and B3^24^. CVA16 genotype C was discovered in Peru^25^, while genotype D was discovered in Peru, France, and China^26, 27^. CVA6 can be categorized into 4 genotypes (A–D). The prototype strain Gdula isolated in the USA in 1949 is classified as genotype A. Genotypes B, C, and D, are subdivided into subgenotypes B1–B2, C1–C2, and D1–3, respectively^28, 29^.

The phylogenetic analyzes of VP1 or whole genome sequences had shown that the co-circulation of multiple subgenotypes of EV-A71, CVA16, and CVA6 in the same outbreaks was frequent and led to the viral genetic recombination events^14, 17^. The co-existence of multiple EV types, genotypes, and subgenotypes might influence genetic diversity and the emergence of new variants. Therefore, this study aimed to investigate EV’s evolutionary dynamics and genetic background, which might affect viral virulence, immune response, vaccine selection, and vaccine development.

## Materials and methods

### Ethics statement

The Human Research Ethics Committee of Thammasat University (Science), (HREC-TUSc), approved this study with the approval number COA No. 136/2562.

### Datasets of Thailand EV-A71, CVA16 and CVA6 VP1 nucleotide sequences

The complete VP1 nucleotide sequences of EV-A71, CVA16, and CVA6 were 891, 891, and 915 nucleotides long, which encode 297, 297, and 305 amino acids, respectively. Here, we examined 385 EV-VP1 sequences collected between 2000 and 2022 from 367 HFMD cases, one asymptomatic HFMD contact adult, one encephalitic pediatric case, and 16 cases without clinical history. This study retrieved these 385 VP1 sequences comprising 228 EV-A71, 33 CVA16, and 124 CVA6 from the NCBI GenBank database (https://ncbi.nlm.nih.gov/) and the Virus Pathogen Database and Analysis Resource (ViPR) (https://legacy.viprbrc.org/brc/home.spg?decorator=vipr). We provided the metadata of the virus names, accession numbers, clinical diagnosis, and geographical locations in Supplementary Table S1. Unfortunately, of 385 VP1 sequences, the geographical locations were available only from 77 VP1 sequences. Nevertheless, these 77 viruses came from 11 provinces in 4 regions (central, northern, northeastern, and western regions) of Thailand.

### Phylogenetic analysis for evolutionary dynamics

The VP1 nucleotide sequences were aligned using Muscle in AliView v1.26 (https://ormbunkar.se/aliview/)^30^. A maximum likelihood (ML) phylogenetic tree was estimated using IQ-TREE (v.1.6.12) under the best-fit nucleotide substitution model (TIM3e + R2 for EV-A71, TNe + I for CVA16 and TNe + G4 for CVA6) determined by ModelFinder as shown in Supplementary Table S2^31^. The ultrafast bootstrap with 1,000 replicates was applied. Root-to-tip regression analysis was made using TempEst v1.5.3 to investigate the temporal signal of the dataset exhibited an association between the divergences from tree root and sampling dates. The time-scaled tree and historical population dynamics including time of the most recent common ancestor (tMRCA) and the rate of evolution (substitution/site/year, s/s/y) were estimated using BEAST package v1.10.4 with a Bayesian Markov chain Monte Carlo (MCMC) approach for phylogenetic reconstruction^32^. The nucleotide substitution model was test by using ModelFinder, and the Bayesian Skygrid model with relaxed molecular clock was employed. The grid was constructed and the population sizes per year were estimated. The MCMC lengths of 50,000,000 generations with sampling every 5000 generations were performed and individually obtained the most effective sample sizes over 200 traced in Tracer v1.7.1. The maximum clade credibility (MCC) tree was determined using TreeAnnotator v1.10.4 and visualized in Figtree v1.4.4 (http://tree.bio.ed.ac.uk/software/figtree). The tMRCA and its 95% highest probability density (95% HPD) were expressed as a year.

## Results

### Thailand enteroviral sequences circulating between 2000 and 2022

As of 18 March 2023, a total of 1,775 EV-A sequences originated in Thailand were available in the public databases. We chose the EV-A71, CVA16, and CVA6 VP1 that contained complete VP1 sequences or, minimally, 90% of the length of the complete sequences and obtained 385 sequences for further genotypic characterization and genetic evolution. These 385 sequences comprised 228 EV-A71, 33 CVA16, and 124 CVA6.

**Figure 1:**
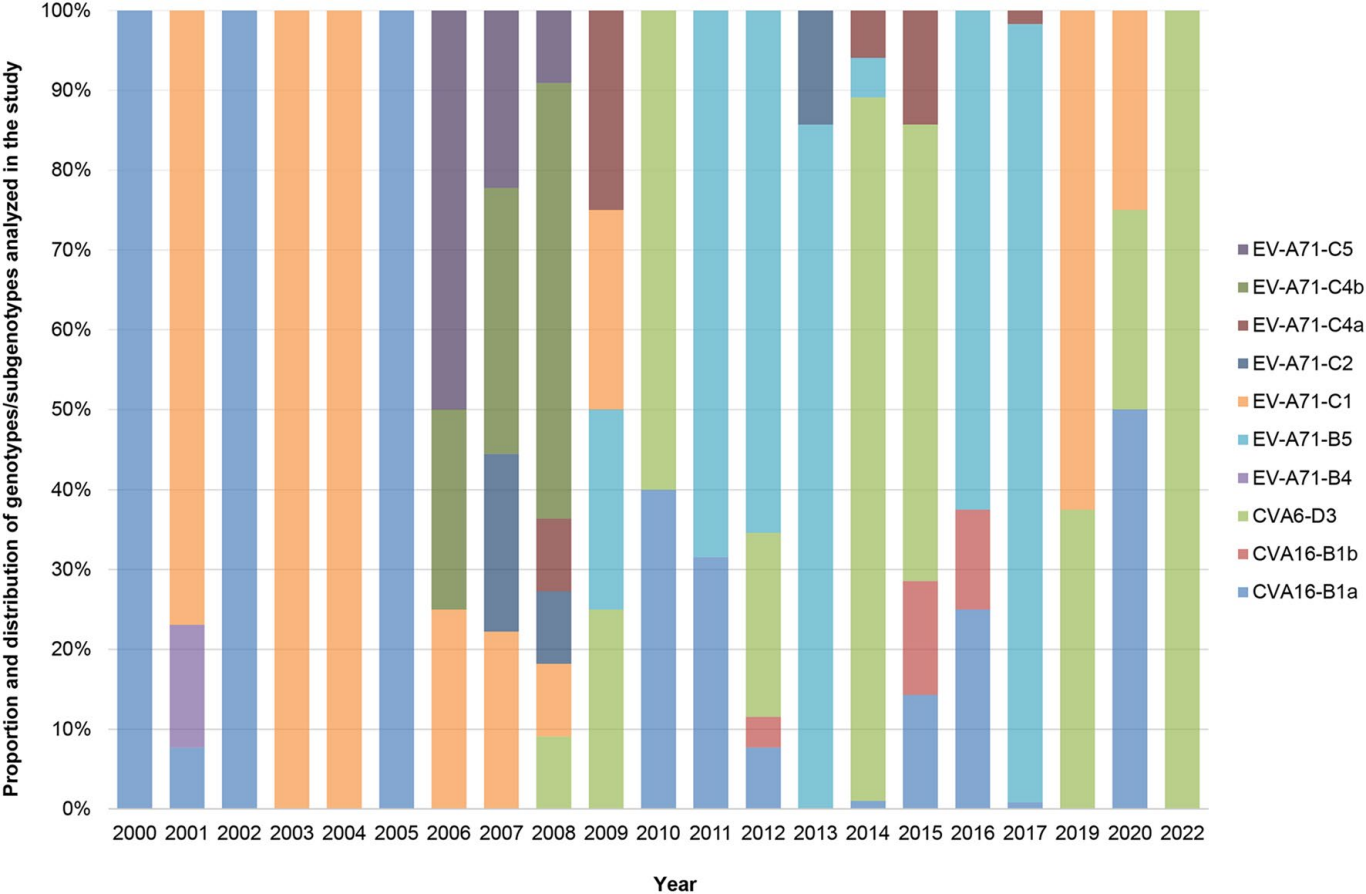
Distribution and proportion of genotypes/subgenotypes of EV-A71, CVA16 and CVA6 Thailand viruses based on complete or at least 90% of VP1 nucleotide sequence length available in databases between 2000 and 2022.

The 228 EV-A71 sequences comprised 164 genotype B and 64 genotype C. The 164 EV-A71 genotype B comprised two subgenotype B4 obtained in 2001 and 162 subgenotype B5 circulating between 2009 and 2017. Among 64 EV-A71 genotype C, 34 sequences belonged to subgenotype C1, four belonged to C2, 11–C4a, 10–C4b, and five to C5. The 33 CVA16 comprised 30 B1a and three B1b subgenotypes. Of 124 CVA6, all belonged to subgenotype D3. Details about these sequences are shown in Supplementary Table S1. The proportion and distribution of enteroviruses by types, genotypes, and subgenotypes each year are shown in Fig. 1.

### Molecular evolution of EV-A71

**Figure 2:**
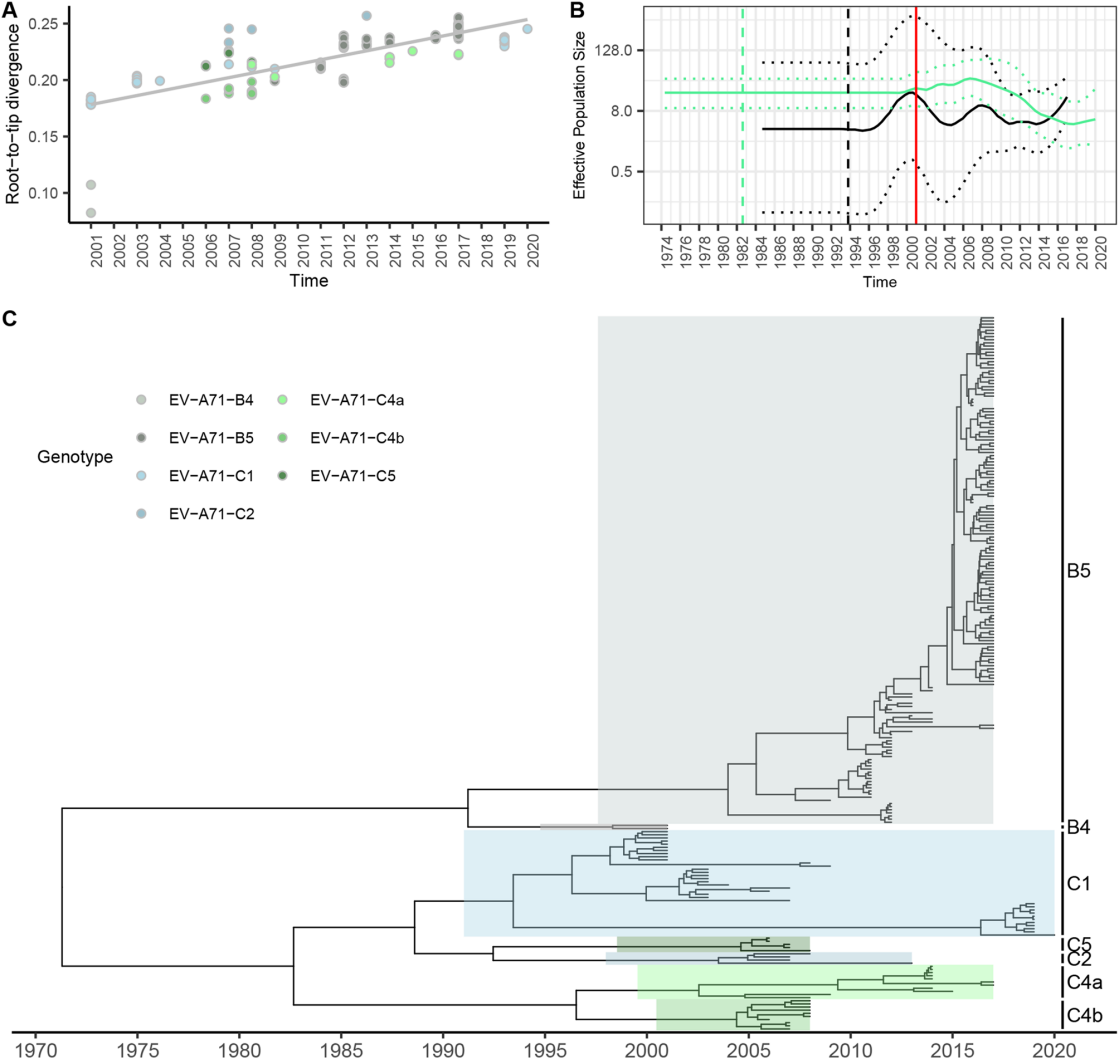
Molecular clock for evolution analysis of EV-A71 genotypes/subgenotypes based on VP1 nucleotide sequences. (**A**) Evolutionary temporal signal by root-to-tip divergence analysis. (**B**) Demographic history of EV-A71 genotypes B and C was inferred by the Bayesian Skygrid model. The timescale in years is indicated in the *x*-axis, and the effective population size is indicated in the *y*-axis. (**C**) Bayesian maximum clade credibility (MCC) phylogenetic tree.

This study analyzed 228 EV-A71 VP1 sequences circulating in Thailand between 2001 and 2020. The root-to-tip divergence analysis showed that EV-A71 B5, C1, C2, C4a, C4b, and C5 subgenotypes had positive temporal signals, except the B4, which exhibited a different divergence from other subgenotypes (Fig. 2A). We further investigated each genotype on the estimated effective population dynamics over time using Bayesian Skygrid analysis (Fig. 2B). The EV-A71 genotype B viruses had wavy fluctuation throughout of 2000 until 2014, and then rapidly increased after 2015 onward. On the other hand, the EV-A71 genotype C exhibited a constant trend since it emerged in 2001 and then slightly decreased until 2020. The tree topology of the whole dataset of EV-A71 VP1 revealed that the viruses were separated into genotypes B and C (Fig. 2C and Supplementary Fig. S1). The tMRCA of genotypes B and C viruses dated at 1993.79 (95% HPD 1984.65–1999.45) and 1982.62 (95% HPD 1974.16–1990.84), respectively (Table 1). The time scale MCC tree showed that B4 shortly circulated in 2001 and disappeared, while B5 emerged later with the estimated tMRCA of 2002.68 (95% HPD 1997.25–2006.83), then expanded and became the major circulating genotype. The estimated mean substitution rate of B5 was 3.53 × 10^–3^ s/s/y (95% HPD 2.74–4.36 × 10^–3^ s/s/y) (Table 1).The EV-A71 genotype C viruses had 5 descending clusters corresponding to the subgenotypes C1, C2, C4a, C4b, and C5. The earliest subgenotype was C1 which shared the tMRCA at 1994.36 (95% HPD 1986.57–1998.12), with the estimated mean substitution rate of 3.41 × 10^–3^ s/s/y (95% HPD 2.32–4.54 × 10^–3^ s/s/y) (Table 1). The EV-A71 C1 circulated in 2001–2009 and 2019–2020. Interestingly, the recent C1 formed a distinct cluster apart from the 2000s cluster. Furthermore, other subgenotypes of EV-A71 including C2, C4a, C4b, and C5 emerged during 2006–2008. The circulation of subgenotypes C4b and C5 was observed for 2–3 years (in 2006–2008), whereas C2 and C4a circulated in 2007–2013 and 2008–2017. Notably, the viral sequences belonging to the same subgenotype, grouped in the same cluster, corresponding to the collected year (Fig. 2C). The estimated tMRCA of C4 viruses was 1996.89 (95% HPD 1984.59–2005.04). The estimated mean substitution rate was 4.02 × 10^–3^ s/s/y (95% HPD 1.82–7.16 × 10^–3^ s/s/y) (Table 1).

**Table 1:** The mean time of the most recent common ancestor (tMRCA), 95% highest probability density (HPD) and estimated the mean substitution rate of Thailand EV-A71, CVA16 and CVA6.

Virus	Genotype/ subgenotype	tMRCA	95% HPD interval	Mean rate (10^–3^ s/s/y)	95% HPD interval (10^–3^ s/s/y)
Enterovirus A71	Genotype B	1993.79	1984.65–1999.45	3.95	3.03–5.04
	Subgenotype B5	2002.68	1997.25–2006.83	3.53	2.74–4.36
	Genotype C	1982.62	1974.16–1990.84	3.44	2.64–4.46
	Subgenotype C1	1994.36	1986.57–1998.12	3.41	2.32- 4.54
	Subgenotype C4	1996.89	1984.59–2005.04	4.02	1.82–7.16
Coxsackievirus A16	Subgenotype B1	1995.86	1993.05–1998.19	4.28	3.20–5.26
Coxsackievirus A6	Subgenotype D3	2007.31	2006.00–2007.99	5.78	4.78–6.8

HPD, highest probability density; s/s/y, substitution/site/year.

### Molecular evolution of CVA16 and CVA6

**Figure 3:**
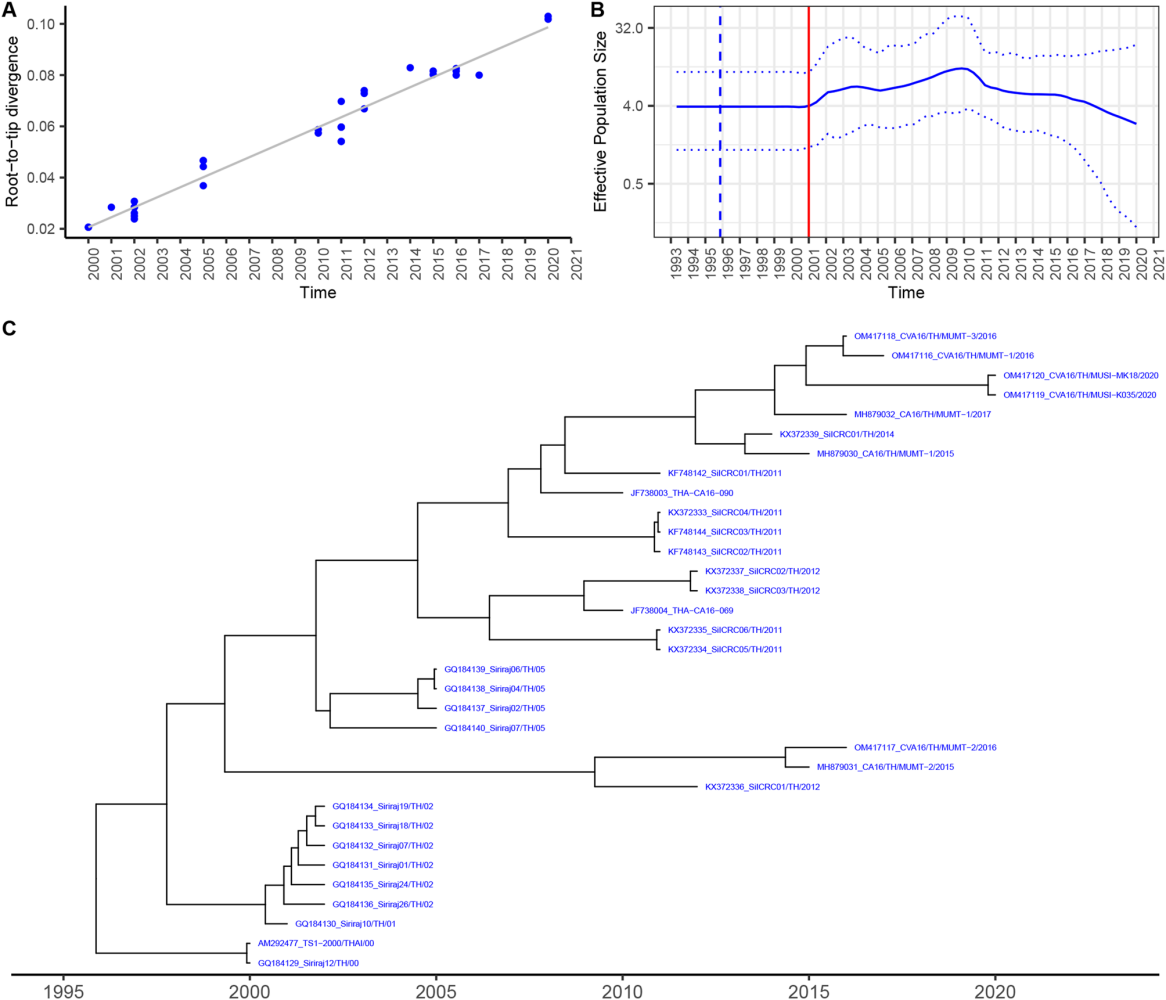
Molecular clock for evolution analysis of CVA16 genotype B1 based on VP1 nucleotide sequences. (**A**) Evolutionary temporal signal by root-to-tip divergence analysis. (**B**) Demographic history of CVA16 genotype B1 was inferred by the Bayesian Skygrid model. The timescale in years is indicated in the *x*-axis, and the effective population size is indicated in the *y*-axis. (**C**) Bayesian maximum clade credibility (MCC) phylogenetic tree.

We phylogenetically analyzed the 33 and 124 VP1 sequences of CVA16 and CVA6, respectively reported in Thailand between 2000 and 2022. The root-to-tip analysis showed that CVA16 had positive temporal signal (Fig. 3A). The estimated effective population dynamics over time using Bayesian Skygrid analysis demonstrated that the CVA16 slightly increased since it emerged in 2000 until 2011, then gradually declined onward (Fig. 3B). The time scale MCC tree showed that CVA16 subgenotype B1a has been constantly circulating for almost two decades; while subgenotype B1b co-circulated with B1a during 2012–2016 (Fig. 3C). The tMRCA of CVA16 genotypes B1 viruses dated at 1995.86 (95% HPD 1993.03–1998.19). The estimated mean substitution rate of CVA16 B1 was 4.28 × 10^–3^ s/s/y (95% HPD 4.78–6.80 × 10^–3^ s/s/y) (Table 1).

**Figure 4:**
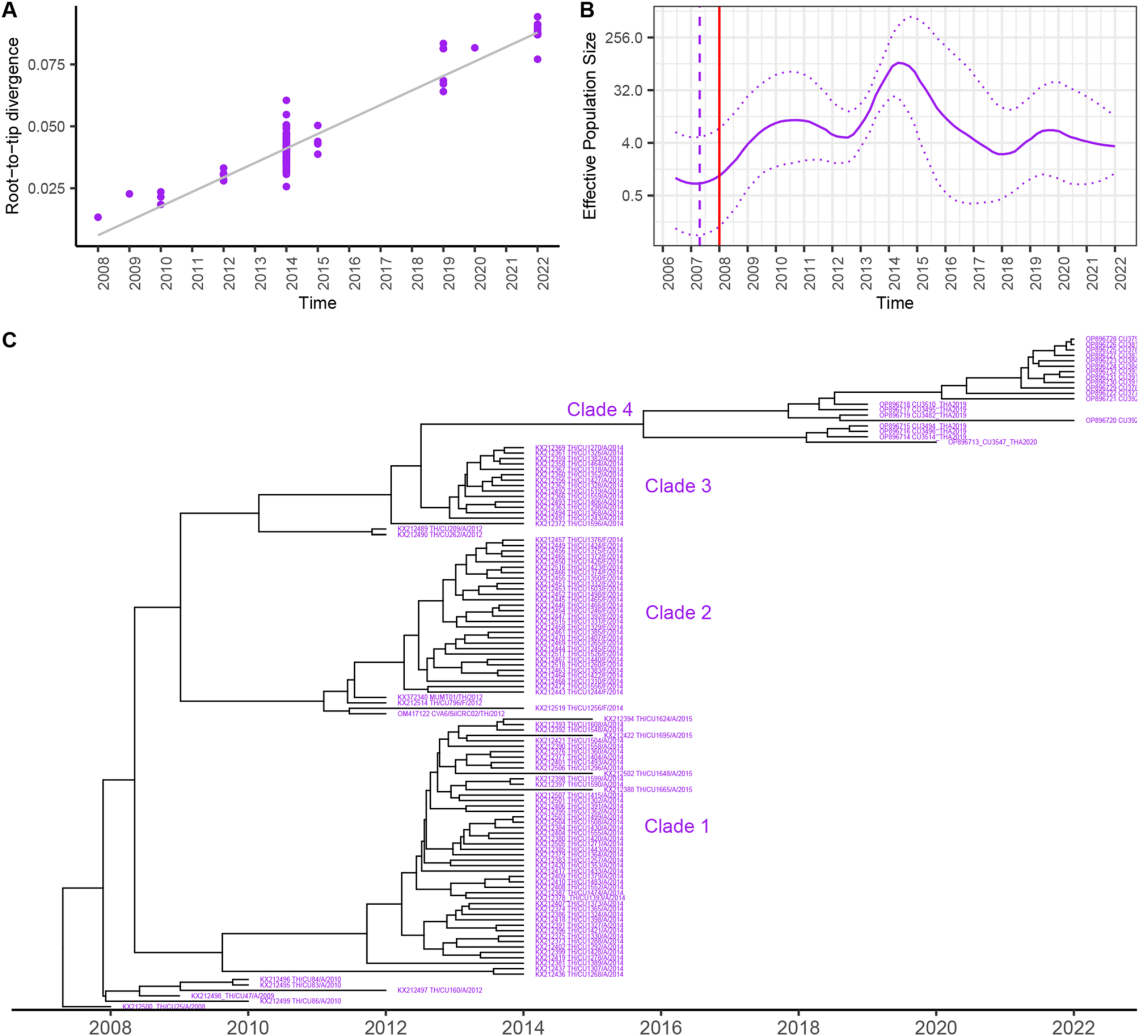
Molecular clock for evolution analysis of CVA6 subgenotype D3 based on VP1 nucleotide sequences. (**A**) Evolutionary temporal signal by root-to-tip divergence analysis. (**B**) Demographic history of CVA6 genotype D3 was inferred by the Bayesian Skygrid model. The timescale in years is indicated in the *x*-axis, and the effective population size is indicated in the *y*-axis. (**C**) Bayesian maximum clade credibility (MCC) phylogenetic tree.

For CVA6, the root-to-tip analysis revealed positive temporal signal (Fig. 4A). The estimated effective population dynamics over time using Bayesian Skygrid analysis demonstrated that the CVA6 exhibited slightly increasing trend since it emerged in 2008, and then rapidly and sharply increased in 2013 through 2015 before subsiding onward (Fig. 4B). The tree topology of the whole dataset of CVA6 VP1 revealed that the viruses belonged to subgenotype D3 with dividing four separated clades (clades 1–4) (Fig. 4C). The tMRCA of CVA6 D3 viruses was dated at 2007.31 (95% HPD 2006.00–2007.99), and the estimated mean substitution rate was 5.78 × 10^–3^ s/s/y (95% HPD 4.78–6.80 × 10^–3^ s/s/y) (Table 1).

### Analysis of VP1 amino acid variation

The complete VP1 amino acid sequences of EV-A71, CVA16, and CVA6 had the actual lengths of 297, 297, and 305 amino acids, respectively. Here, we comprehensively analyzed the deduced complete VP1 amino acid sequences of EV-A71 (n = 219), CVA16 (n = 33), and CVA6 (n = 47) for their genetic variation using the ClustalW multiple alignment and sequence identity matrix application in BioEdit version 7.0.4.1. The analysis showed that non-synonymous mutations that resulted in amino acid changes were scattered across the VP1 region. EV-A71 strains encompassing subgenotypes B4, B5, C1, C2, C4a, C4b, and C5 harbored 65 amino acid substitution positions, with the variation at a range of 0.4–9.5% (90.5–99.6% identities). The intra-subgenotypic variation ranged from 0 to 8.5% (91.5–100% identities), while the inter-subgenotypic variations were 0.4–9.5% (90.5–99.6% identities). In addition, there were 11 amino acid substitution positions with a range of 0–2.9% (97.1–100% identities) observed among CVA16 strains, while, there were 32 amino acid substitution positions with a range of 0–4% (96.0–100% identities) observed among CVA6 strains. The results are shown in Supplementary Table S3.

## Discussion

This study reported the genetic evolution of the Thailand EV-A71, CVA16, and CVA6 based on the VP1nucleotide sequences deposited in the databases between 2000 and 2022. The VP1 region is hypervariable and the major neutralizing domain of enteroviruses. The positive selection at VP1 immunogenic site made enteroviruses rapidly continue to evolve. Therefore, VP1 was recommended for enterovirus genotyping and studying genetic evolution^33, 34^. A minimum length of 350 nucleotides was required for virus typing and molecular epidemiology, while the complete VP1 sequence was required to assign new enterovirus genotypes^34^. Hence, we conducted a Bayesian phylogenetic reconstruction and molecular clock analysis using a complete VP1 region, or at least 90% of VP1 length.

Between 2000 and 2017, we previously reported that multiple EV-A71 genotypes/subgenotypes emerged, circulated, and faded out, with intra- or inter-genotypic shift overtimes^4, 15^. Likewise, the dynamics of genotype/subgenotype replacements have been documented in detail in several countries, e.g., China, Singapore, Malaysia, Taiwan, Vietnam, and Japan, during the past two decades^2, 4, 35, 36^. Currently, the most common subgenotypes circulated in Thailand are B5, C1, and C4 in EV-A71^15, 18, 37, 38^, while C4 is the most common in China and Vietnam^39, 40^. The EV-A71 vaccines produced in China are prepared from the C4 subgenotype^41^.

The present study found that EV-A71 genotypes B and C co-circulating since 2001 in Thailand showed the estimated tMRCA of 1993.79 and 1982.62, respectively. This study suggested that EV-A71 was introduced and cryptically circulated in Thailand for years before the MOPH officially reported the HFMD cases in 2001. The EV-A71 C1 virus had been circulating in Thailand from 2002 to 2009, and multiple lineages of recombinant C1 re-emerged and circulated during 2019 to 2020. Those re-emerging C1 virus was genetically related to the variants detected in Europe and showed the estimated substitution rate of 4.38 × 10^–3^ s/s/y)^38^. In addition, the introduction of EV-A71 subgenotypes C4a and C4b into Thailand was first documented in 2008 and 2006, respectively; with the estimated tMRCA of 1996.89 for EV-A71 C4 virus. Indeed, the EV-A71 C4 virus was the most common and has persistently circulated in China since 1998 up to the present^42, 43^. The EV-A71 C4 virus also circulated in Taiwan in 1998, Japan in 2002, Vietnam in 2005, and Australia in 2004^2, 4, 35^. We first discovered the EV-A71 C5 in 2006, and the virus co-existed with C4b for 3 years between 2006 and 2008. In the meantime, the intra-genotypic replacement from EV-A71 C4b and C5 to C4a began in 2008. Similarly, the emergence of the EV-A71 B5 virus was first recognized in 2006 and has been co-circulated with C4a in HFMD patients since 2008 until the present^15^. The current HFMD-associated-EV-A71 subgenotypes in Thailand comprised the subgenotypes C1, B5, and C4a^18, 37, 38^. The analysis of genetic evolution and epidemiological population dynamics demonstrated that the cyclical pattern of EV-A71 epidemics in Thailand was about 2–3 years, which was in line with those observed in several countries in the Asia–Pacific region^3, 4, 44, 45^.

CVA16 and CVA6 co-circulated with EV-A71 during HFMD outbreaks in several countries^3, 6, 10^. Occasionally, these two viruses were more prominent than EV-A71, varying by year and region studied. In Thailand, all CVA16 clustered in genotype B1 (predominantly B1a and B1b)^15^. No CVA16 subgenotypes B1c and B2 were found in Thailand, while those have been sporadically reported in China, Malaysia, France and Japan^5^. Our previous study demonstrated that CVA16 subgenotype B1a had been persistently circulating in Thailand for two decades, suggesting that the genetic diversity of CVA16 was less than EV-A71. The CVA16 Thailand viruses were phylogenetically related to those reported in China and Vietnam^15^. Likewise, the CVA16 B1 was also the predominant genotype for HFMD cases in China from 2000 to 2018^5, 6, 46^, and it is a recommended candidate for vaccine development^47^. On the other hand, HFMD outbreaks caused by CVA6 were more frequent in several countries in Europe, Northern America, and Asia^48^. Similarly, in Thailand, CVA6 virus had been identified as the principal etiologic agent responsible for HFMD outbreaks in 2012^16^. After the outbreaks subsided, CVA6 became one of the common HFMD etiologic agents, co-circulating with EV-A71 and CVA16. Although the CVA6 comprised multiple genotypes (A–D) and subgenotypes (B1–B2, C1–C2, and D1–3), only subgenotype D3 is present in Thailand. This study demonstrated that our CVA6 virus was phylogenetically classified into four distinct clades (clades 1–4), suggesting multiple introductions of the viruses from different origins^17^.

Mutation and recombination, which drives the evolutionary process to significant genotypic and phenotypic variability, are common in enteroviruses^49^. The genetic changes leading to antigenic shifts might be responsible for causing outbreaks. Non-synonymous mutations in VP1 that lead to amino acid changes were common among enteroviruses. This study showed the mean rates of VP1 nucleotide substitutions in the range of 3.41–5.78 × 10^–3^ s/s/y among EV-A71, CVA16 and CVA6, which correspond with the other reports^6, 17, 33, 38^. As VP1 plays an essential role in protective immunity, the genetic change at the critical site in VP1 might yield the immune escape mutants that affect the vaccine efficacy.

In addition to VP1 mutation, genetic recombination was an essential mechanism for intra-genomic rearrangements for genetic diversity and evolution. We have previously phylogenetically analyzed complete genome sequences and individual regions of the genome (including CDS, P1, P2, P3, VP1, VP2, VP3, VP4, 2A, 2B, 2C, 3A, 3B, 3C, and 3D regions) of EV-A71, CVA16^14^, and CVA6 for genetic recombination [unpublished data]. The result demonstrated several recombinants among our study viruses: EV-A71, CVA16, and CVA6, in which we identified the P2 and P3 regions as the recombination breakpoints.

In conclusion, this study investigated the evolutionary dynamics and genetic history of EV-A71, CVA16, and CVA6, providing insight into a better understanding of molecular epidemiology. Multiple subgenotypes of EV-A71, CVA16, and various genetically distinct CVA6 clades have been introduced and co-circulated in Thailand. Genotypic/subgenotypic replacement, fluctuation and cyclical pattern of those viruses were observed along the two decades of observation. It is implied that the virus continues to evolve and potentially causes large-scale outbreaks in naïve-immune population. In addition, the emergence of new genotype/subgenotype, the intra- and inter-genotypic replacements, and the introductions of new variants generated by spontaneous mutation and genetic recombination raise the challenges for vaccine development, selection, and vaccination policy. The laboratory-based monitoring and epidemiological surveillance for genetic changes and evolutionary studies are necessary.

### Supplementary Information

Supplementary Figure S1.

Supplementary Legends.

Supplementary Table S1.

Supplementary Table S2.

Supplementary Table S3.

## Data Availability

The raw data are available in the Supplementary files. The dataset generated during analysis are available upon reasonable request to the corresponding author.

## References

[1] World Health Organization. *Hand, foot and mouth disease (HFMD)*. Accessed 18 December 2022, https://www.who.int/westernpacific/emergencies/surveillance/archives/hand-foot-and-mouth-disease (2022)..

[2] Yip CC, Lau SK, Woo PC, Yuen KY (2013). Human enterovirus 71 epidemics: what's next?. Emerg. Health Threats. J..

[3] Ji T (2019). Surveillance, epidemiology, and pathogen spectrum of hand, foot, and mouth disease in mainland of China from 2008 to 2017. Biosaf. Health..

[4] Puenpa J, Wanlapakorn N, Vongpunsawad S, Poovorawan Y (2019). The history of enterovirus A71 outbreaks and molecular epidemiology in the Asia-Pacific region. J. Biomed. Sci..

[5] Chen X (2013). Molecular epidemiology of coxsackievirus A16: intratype and prevalent intertype recombination identified. PLoS ONE..

[6] Han Z (2020). Genomic epidemiology of coxsackievirus A16 in mainland of China, 2000–18. Virus. Evol..

[7] Centers for Disease Control and Prevention. Hand, foot, and mouth disease (HFMD). Accessed 18 December 2022, https://www.cdc.gov/hand-foot-mouth/about/signs-symptoms.html (2022).

[8] Cox JA, Hiscox JA, Solomon T, Ooi MH, Ng LFP (2017). Immunopathogenesis and virus-host interactions of enterovirus 71 in patients with hand, foot and mouth disease. Front. Microbiol..

[9] Oberste MS, Peñaranda S, Maher K, Pallansch MA (2004). Complete genome sequences of all members of the species Human enterovirus A. J. Gen. Virol..

[10] Bian L (2015). Coxsackievirus A6: A new emerging pathogen causing hand, foot and mouth disease outbreaks worldwide. Expert. Rev. Anti Infect. Ther..

[11] Bureau of Epidemiology, Department of Disease Control, MOPH, Thailand. Hand, foot and mouth (HFM) disease. Accessed 20 July 2023http://doe.moph.go.th/surdata/disease.php?dcontent=old&ds=71 (2023).

[12] Bureau of Epidemiology, Department of Disease Control, MOPH, Thailand. Hand, foot and mouth disease (in Thai). Accessed 20 July 2023 http://odpc9.ddc.moph.go.th/SRRTcenter/dataL3_241.pdf (2007).

[13] Verma S (2021). Hand, foot, and mouth disease in Thailand: a comprehensive modelling of epidemic dynamics. Comput. Math. Methods. Med..

[14] Noisumdaeng P (2018). Complete genome analysis demonstrates multiple introductions of enterovirus 71 and coxsackievirus A16 recombinant strains into Thailand during the past decade. Emerg. Microbes Infect..

[15] Noisumdaeng P (2019). Longitudinal study on enterovirus A71 and coxsackievirus A16 genotype/subgenotype replacements in hand, foot and mouth disease patients in Thailand, 2000–2017. Int. J. Infect. Dis..

[16] Puenpa J (2013). Hand, foot, and mouth disease caused by coxsackievirus A6, Thailand, 2012. Emerg. Infect. Dis..

[17] Puenpa J (2022). Evolutionary and genetic recombination analyses of coxsackievirus A6 variants associated with hand, foot, and mouth disease outbreaks in Thailand between 2019 and 2022. Viruses.

[18] Sittikul P (2023). Diversity of human enterovirus co-circulations in five kindergartens in Bangkok between July 2019 and January 2020. Viruses..

[19] Yuan J (2018). Enterovirus A71 proteins: Structure and function. Front. Microbiol..

[20] Bessaud M (2014). Molecular comparison and evolutionary analyses of VP1 nucleotide sequences of new African human enterovirus 71 isolates reveal a wide genetic diversity. PLoS ONE..

[21] Majumdar M (2018). Environmental surveillance reveals complex enterovirus circulation patterns in human populations. Open Forum. Infect. Dis..

[22] Liu Y (2022). A novel subgenotype C6 enterovirus A71 originating from the recombination between subgenotypes C4 and C2 strains in mainland China. Sci. Rep..

[23] Schmidt NJ, Lennette EH, Ho HH (1974). An apparently new enterovirus isolated from patients with disease of the central nervous system. J. Infect. Dis..

[24] Chen L (2019). Molecular surveillance of coxsackievirus A16 reveals the emergence of a new clade in mainland China. Arch. Virol..

[25] Carrion G (2016). Molecular epidemiology of coxsackievirus A16 strains from four sentinel surveillance sites in Peru. Int. J. Infect. Dis..

[26] Wang J (2018). The emergence and spread of one Coxsackievirus A16 genogroup D novel recombinant strain that caused a clustering HFMD outbreak in Shanghai, China, 2016. Emerg. Microbes Infect..

[27] Hassel C (2017). Phylogeography of coxsackievirus A16 reveals global transmission pathways and recent emergence and spread of a recombinant genogroup. J. Virol..

[28] Song Y (2020). Genetic recombination in fast-spreading coxsackievirus A6 variants: a potential role in evolution and pathogenicity. Virus. Evol..

[29] Liu H (2021). Characterization of coxsackievirus A6 strains isolated from children with hand, foot, and mouth disease. Front. Cell. Infect. Microbiol..

[30] Larsson A (2014). AliView: A fast and lightweight alignment viewer and editor for large datasets. Bioinformatics.

[31] Trifinopoulos J, Nguyen LT, von Haeseler A, Minh BQ (2016). W-IQ-TREE: A fast online phylogenetic tool for maximum likelihood analysis. Nucleic Acids Res..

[32] Suchard MA (2018). Bayesian phylogenetic and phylodynamic data integration using BEAST 1.10. Virus. Evol..

[33] Tee KK (2010). Evolutionary genetics of human enterovirus 71: Origin, population dynamics, natural selection, and seasonal periodicity of the VP1 gene. J. Virol..

[34] Harvala H (2018). Recommendations for enterovirus diagnostics and characterisation within and beyond Europe. J. Clin. Virol..

[35] Huang SW, Cheng D, Wang JR (2019). Enterovirus A71: Virulence, antigenicity, and genetic evolution over the years. J. Biomed. Sci..

[36] Geoghegan JL (2015). Phylodynamics of enterovirus A71-associated hand, foot, and mouth disease in Viet Nam. J. Virol..

[37] Puenpa J, Auphimai C, Korkong S, Vongpunsawad S, Poovorawan Y (2018). Enterovirus A71 infection, Thailand, 2017. Emerg. Infect. Dis..

[38] Puenpa J (2021). Genetic diversity and evolution of enterovirus A71 subgenogroup C1 from children with hand, foot, and mouth disease in Thailand. Arch. Virol..

[39] Sun H, Gao M, Cui D (2020). Molecular characteristics of the VP1 region of enterovirus 71 strains in China. Gut. Pathog..

[40] Romanenkova NI (2023). Enterovirus 71-associated infection in South Vietnam: Vaccination is a real solution. Vaccines.

[41] Li ML, Shih SR, Tolbert BS, Brewer G (2021). Enterovirus A71 vaccines. Vaccines.

[42] Zhou J, Shi Y, Miao L, Zhang C, Liu Y (2021). Molecular epidemiology and recombination of enterovirus A71 in mainland China from 1987 to 2017. Int. Microbiol..

[43] Zhang Y (2013). Complete genome analysis of the C4 subgenotype strains of enterovirus 71: Predominant recombination C4 viruses persistently circulating in China for 14 years. PLoS ONE..

[44] NikNadia N (2016). Cyclical patterns of hand, foot and mouth disease caused by enterovirus A71 in Malaysia. PLoS Negl. Trop. Dis..

[45] Takahashi S (2018). Epidemic dynamics, interactions and predictability of enteroviruses associated with hand, foot and mouth disease in Japan. J. R. Soc. Interface..

[46] Guo J (2022). Epidemiology of hand, foot, and mouth disease and the genetic characteristics of Coxsackievirus A16 in Taiyuan, Shanxi, China from 2010 to 2021. Front. Cell. Infect. Microbiol..

[47] Cheng D (2022). Genetic and cross neutralization analyses of coxsackievirus A16 circulating in Taiwan from 1998 to 2021 suggest dominant genotype B1 can serve as vaccine candidate. Viruses.

[48] Zhu P (2023). Current status of hand-foot-and-mouth disease. J. Biomed. Sci..

[49] Muslin C, Mac Kain A, Bessaud M, Blondel B, Delpeyroux F (2019). Recombination in enteroviruses, a multi-step modular evolutionary process. Viruses.

