# Supplementary figures and images for: Molecular evolutionary dynamics of enterovirus A71, coxsackievirus A16 and coxsackievirus A6 causing hand, foot and mouth disease in Thailand, 2000–2022
Source: Sci Rep. 2023 Oct 13;13:17359. doi: 10.1038/s41598-023-44644-z (PMC10576028; doi:10.1038/s41598-023-44644-z)

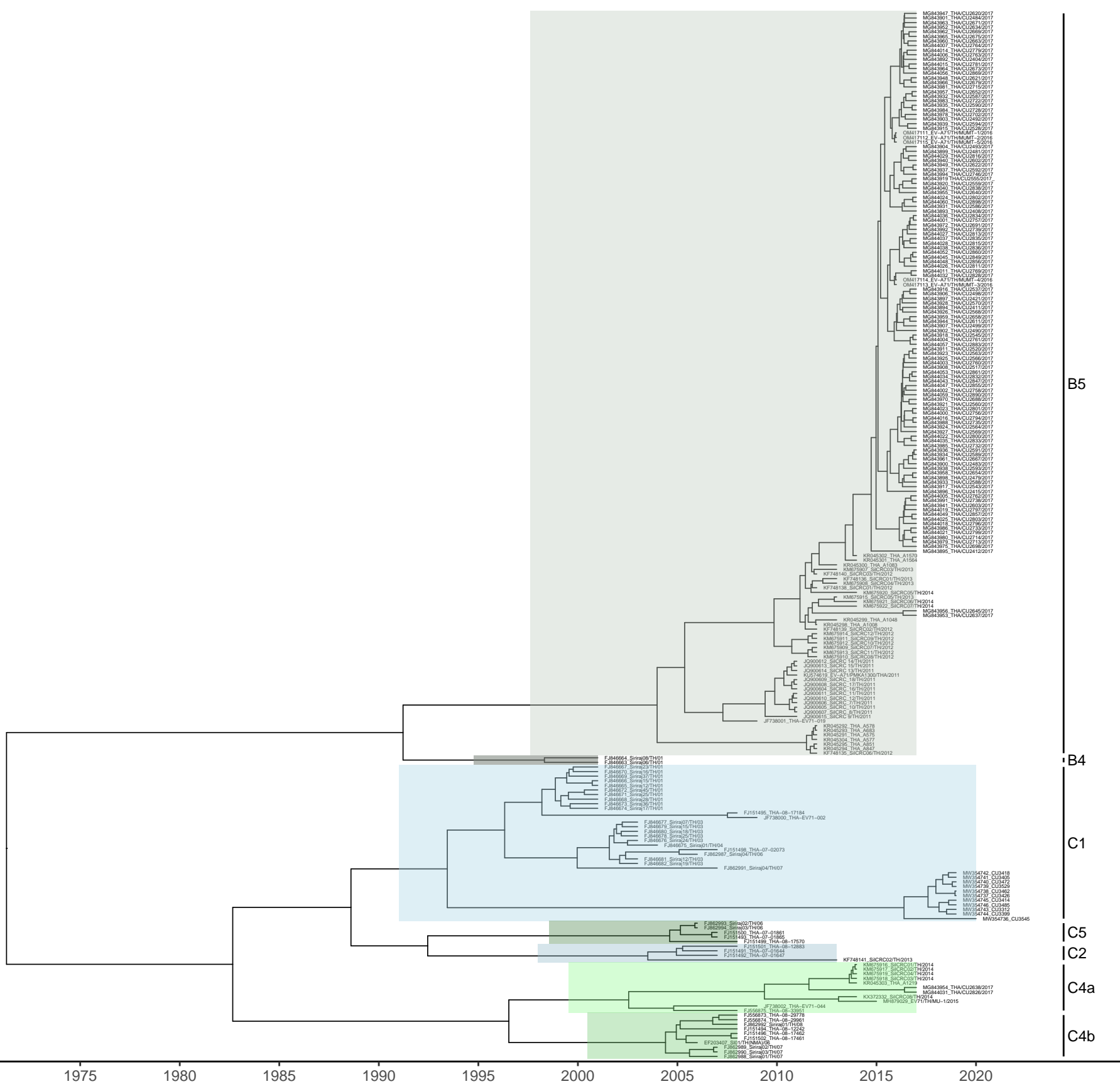

Supplement: Supplementary file 1 — Supplementary Figure S1. [file 41598_2023_44644_MOESM1_ESM.pdf]
