# Supplementary material for: Molecular evolutionary dynamics of enterovirus A71, coxsackievirus A16 and coxsackievirus A6 causing hand, foot and mouth disease in Thailand, 2000–2022
Source: Sci Rep. 2023 Oct 13;13:17359. doi: 10.1038/s41598-023-44644-z (PMC10576028; doi:10.1038/s41598-023-44644-z)
Supplement: Supplementary file 2 — Supplementary Legends. [file 41598_2023_44644_MOESM2_ESM.docx]

**Supplementary information**

**Supplementary Table S1.** Datasets of EV-A71, CVA16 and CVA6 Thailand isolates analyzed in the study.

**Supplementary Table S2.** The best fit nucleotide substitution model of EV-A71, CVA16 and CVA6 determined by MoldelFinder.

**Supplementary Table S3.** VP1 amino acid sequence analyzes among EV-A71, CVA16 and CVA6 Thailand isolates for detecting amino acid variability and similarity

**Supplementary Figure S1.** Bayesian maximum clade credibility (MCC) phylogenetic tree of EV-A71 with GenBank accession number and virus name labelling.
